# The Power of the Penny

**Published:** 1889-01-19

**Authors:** 


### The Hospital

A WEEKLY INSTITUTIONAL JOURNAL OF

**Science, Medicine, Nursing, and Philanthropy.**

*For Hospitals, Asylums, Medical Practitioners, Students, Nurses, Families, Parishes, Congregations, and the Charitable Public.*

VOL. V. No. 121.] [JANUARY 19, 1889.

### THE POWER OF THE PENNY.

THE pen is a mighty power, and so is the penny. In olden times "Peter's pence" often replenished the exhausted coffers of the Popes, and in our own day the vast organisation known as "Wesleyan Methodism," may be said to repose entirely on pillars of pence. Co-operation is the antithesis of warfare, not of individualism. Wise co-operation and just individualism are twin sisters, which together will work out the profoundest problems of human life and destiny. When men are agreed in mind they can join hands and accomplish anything which lies within the bounds of possibility; but if minds are at strife no power on earth can bring about real union in action. Strife is madness. The man of discord is more dangerous than the lunatic. In singleness of eye and unity of purpose lie a success and a happiness which can be reached in no other way whatever.

The supporters of hospitals are entirely agreed that they have a great cause in hand. But the necessities of the sick poor are always threatening to outstrip the means of relief; and hence arises the frequent demand for new methods of supplying "ways and means." Just now a method is under consideration which, though not new, has for some people more than the charm of novelty. It is the application of the "penny-a-week" system to the raising of funds for hospitals. The main facts connected with the new proposal are briefly these: Mr. A. C. P. Coote, Secretary of the Lock Hospital, Harrow Road, has written two letters to the *Times*. Therein he repeats the familiar statement that the annual income of the London hospitals is too small by at least a hundred thousand pounds. Five hundred thousand pence per week would amount to a hundred and eight thousand pounds in the year. There are seven hundred and fifty thousand workmen in London. If two-thirds of these will give a penny a-week each, there will be the five hundred thousand weekly pence and the hundred and eight thousand yearly pounds, and so the difficulty is got over *per saltum* and at once. The scheme looks the essence of simplicity; and it is not surprising that Lord Mayor Whitehead, who has a keen eye to business, should have been taken with it, and should have promised its proposer all the aid that a meeting at the Mansion House can give. Such a meeting will be held on the 29th instant, and it may be hoped that all who have the interests of the medical charities at heart will be present.

The idea of asking from working men systematic and periodical contributions to the funds of hospitals is excellent. Indeed it is so good that every possible care must be taken lest there should be any bungling in carrying it out, and so the scheme should be ruined. An organisation of some kind will be needed; and an organisation which can deal efficiently and inexpensively with the whole of the workmen of London can neither be created nor evolved by a stroke of the pen, or even by speeches at the Mansion House. The plan is vast, thorny, and difficult; and its proposers need all the light and guidance which experience and judgment can suggest.

In several towns in the North of England, and particularly at Leeds and Sunderland, the problem of working men's contributions has been solved in a very successful way. At Leeds, Mr. Alderman Spark, and at Sunderland, Mr. A. W. Webster are identified with the movement, and have both expounded their methods of working at some length in the pages of THE HOSPITAL. The workshop is taken as the starting point at Leeds, and the employer, with his heads of departments and foremen, are held to be the natural officials for the collection of the workmen's subscriptions. At the outset it is usual for the employer to call a meeting of all hands at some spare hour, or, better still, to stop work for half an hour before the dinner hour, in order that a meeting may be held. Then the scheme is propounded to the men, and each of them is asked whether he is willing to give a stipulated sum per week, and how much. He is not asked to give so much as even a penny if he feels he cannot afford that sum. He may give a halfpenny or a farthing. On the other hand, if he be a man with good wages and a small family he may give twopence, or threepence, or even more. Books are kept in which each man's promise is entered, and the amount is deducted by the master or cashier when the wages are paid. Some member of the firm or superior employé is made workshop treasurer, and he is responsible for the payment of a sum equivalent to all the promises of the book to a general treasurer at stated periods. The general treasurer, who at Leeds is Mr. Alderman Spark, in his turn pays over the totals from the various workshop centres to the hospital authorities. This scheme is simple, inexpensive, and satisfactory in a high degree. Very much the same methods are adopted in Sunderland, with special adaptations to the necessities of a seaport town. There, not only the workshops, but all British ships entering the harbour, and particularly native vessels, are included in the contributory system, and gladly take their share in what is felt to be an admirable work. The proof of the excellence of these methods is to be found in the results. At Leeds, where the population numbers about three hundred thousand, the workshop collections in 1887 amounted to a little over £3,692. If like methods were adopted in London with like results the London workmen's contributions would leap up at once to more than £60,000. The success at Sunderland has been even more marked than at Leeds. Sunderland has a population of a little more than one hundred thousand. There the amount raised by the workmen's systematic contributions reached the remarkable total of £2,469, exclusive of a sum of £400 raised by a workmen's bazaar. If London, by adopting a method similar to that employed in Sunderland, should produce similar results, the splendid total of £120,000 would be added to the annual income of the metropolitan hospitals by the workmen and sailors alone.

Now these examples cannot be passed by with indifference at the Mansion House meeting, or in any future consideration of a practical scheme. The proposers of the penny-a-week system for London have of course a plan of their own, which they think will work satisfactorily. It will be well to look at their proposals side by side with the methods in actual practice at Leeds and Sunderland. In London it is proposed to establish a local committee in every Parliamentary district, in connexion with the Hospital Saturday movement. What does that imply? Thirty separate committees, with thirty separate offices and thirty separate paid secretaries! At Leeds and Sunderland no such committees, offices, and secretaries are found necessary, for the simple reason that in each workshop and factory the master and his chief employés act as a committee, the factory or workshop is the office, and the master or employé who acts as treasurer is also the unpaid secretary. What an enormous saving is here effected! If it be said that London is so much larger than Leeds or Sunderland, that different methods must be adopted, an answer is at once furnished by the experience of the Hospital Sunday Fund. In the management of that fund one central organisation and office and one paid secretary are found amply sufficient for every practical purpose. Locally each church and chapel manages its own fund without the cost of a single penny to the central organisation. What the church or chapel, with its clergyman and lay officials, is to the Sunday Fund, that must the workshop with its master and principal employés be to the new workmen's fund. This would be simplicity itself—it would be inexpensiveness itself; and if precedent and experience may be relied upon, it would be as completely successful as it would be simple and inexpensive.

The only point that remains to be considered, and it is a point of supreme importance, is, how is such a vast and attractive scheme to be successfully carried out? Now no one must be timid or mealy-mouthed in discussing this initial question. The potency of workmen's contributions in the metropolis is so great and splendid that it will be unpardonable and absurd to wreck the scheme by placing it in incompetent hands. Every man of experience must speak, and speak plainly; and every man of inexperience must keep an open mind. There must be no considering how this or that individual will be affected by any particular proposal. One consideration, and one alone, must be uppermost in all minds, and that consideration must be how to induce every London workman to take his share in the great work, and how to bring every penny of his hard-earned contribution, unclipped and undiminished, to the general fund of the metropolitan charities. The opportunity for which this journal has long laboured seems to be now approaching with rapidity and certainty. It remains to be seen, whether among the thousands of hospital supporters in the metropolis there is the statesmanlike capacity to use it in the broadest, wisest, and most successful way.

